# Synthesis, Characterization, and BSA-Binding Studies of Novel Sulfonated Zinc-Triazine Complexes

**DOI:** 10.1155/2018/7563820

**Published:** 2018-02-18

**Authors:** Nalin Abeydeera, Inoka C. Perera, Theshini Perera

**Affiliations:** ^1^Department of Chemistry, University of Sri Jayewardenepura, Nugegoda, Sri Lanka; ^2^Department of Zoology and Environmental Science, University of Colombo, Colombo, Sri Lanka

## Abstract

Four Zn(II) complexes containing a pyridyl triazine core (*L*1 = 3-(2-pyridyl)-5,6-di(2-furyl)-1,2,4-triazine-5′,5″-disulfonic acid disodium salt and *L*2 = 3-(2-pyridyl)-5,6-diphenyl-1,2,4-triazine-4′,4″-disulfonic acid sodium salt) were synthesized, and their chemical formulas were finalized as [Zn(L1)Cl_2_]·5H_2_O·ZnCl_2_ (**1**), [Zn(L1)_2_Cl_2_]·4H_2_O·2CH_3_OH (**2**), [Zn(L2)Cl_2_]·3H_2_O·CH_3_OH (**3**), and [Zn(L2)_2_Cl_2_] (**4**). The synthesized complexes are water soluble, making them good candidates for biological applications. All four complexes have been characterized by elemental analysis and ^1^H NMR, IR, and UV-Vis spectroscopy. The IR stretching frequency of N=N and C=N bonds of complexes **1**–**4** have shifted to lower frequencies in comparison with free ligands, and a bathochromic shift was observed in UV-Vis spectra of all four complexes. The binding studies of ligands and complexes **1**–**4** with bovine serum albumin (BSA) resulted binding constants (*K*_b_) of 3.09 × 10^4^ M^−1^, 12.30 × 10^4^ M^−1^, and 16.84 × 10^4^ M^−1^ for ferene, complex **1**, and complex **2**, respectively, indicating potent serum distribution via albumins.

## 1. Introduction

The potential use of zinc complexes as antidiabetic insulin mimetics [1], antimicrobial [2], and anticancer agents [3] have garnered a renewed interest in such complexes among other applications, such as serving as tumor photo sensitizers [4], radioprotective agents [5], and antidandruff agents (Zn pyrithione-ZPT) [6]. Our interest in sulfa drug moieties has been fueled by the fact that they possess a wide range of pharmaceutical applications [7]. Of particular interest are 1,2,4-triazine derivatives because they have been reported to possess many biological activities such as kinase inhibition [8], antihypertensivity [9], antimicrobial [10], anticancer [11], anti-HIV [12, 13], and anti-inflammatory activities [8].

**Figure 1:**
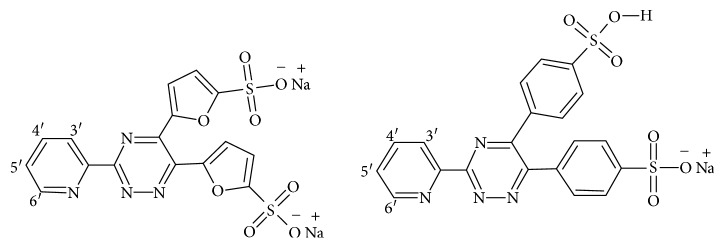
Chemical structures of 3-(2-pyridyl)-5,6-di(2-furyl)-1,2,4-triazine-5′,5″-disulfonic acid disodium salt (ferene: L1) (left) and 3-(2-pyridyl)-5,6-diphenyl-1,2,4-triazine-4′,4″-disulfonic acid sodium salt (ferrozine: L2) (right).

Novel polyanionic sulfonated aromatic synthetic platinum chelates were preliminarily evaluated for their HIV-1 virucidal activity, due to the presence of sulfonated aromatic groups and metals in the most active members [14, 15]. Furthermore, we recently reported that rhenium tricarbonyl complexes of ferene and ferrozine have exhibited the potential to be used as biological imaging agents [16]. (Chemical structures of ferene and ferrozine are illustrated in Figure 1.) However, to the best of our knowledge, no reports exist of zinc complexes of sulfonated 1,2,4-triazine derivatives.

It is noteworthy that although zinc complexes bearing the 5,6-diphenyl-3, 2-pyridyl-1 2,4-triazine ligand were first synthesized more than a decade ago [17, 18], biological studies have been reported for only one such complex, albeit only recently [19]. Therefore, our goal has been to synthesize zinc complexes bearing sulfonated pyridyl triazine derivatives and to assess their interaction with biological targets.

Being the most abundant protein in blood, serum albumin maintains the colloid osmotic pressure, while playing a major role in transport and sustained release of many biomolecules such as steroids, fatty acids, and hormones. Serum albumin also serves as a carrier protein for drug molecules [20]. Small molecule interaction with serum albumin is thus exploited in pharmaceutical research, where affinity to albumins is indicative of drugs with high serum distribution [21]. Through the interaction between bovine serum albumin (BSA), an analog of human serum albumin, and the novel compounds, we seek to investigate their pharmacokinetic associations.

**Figure 2:**
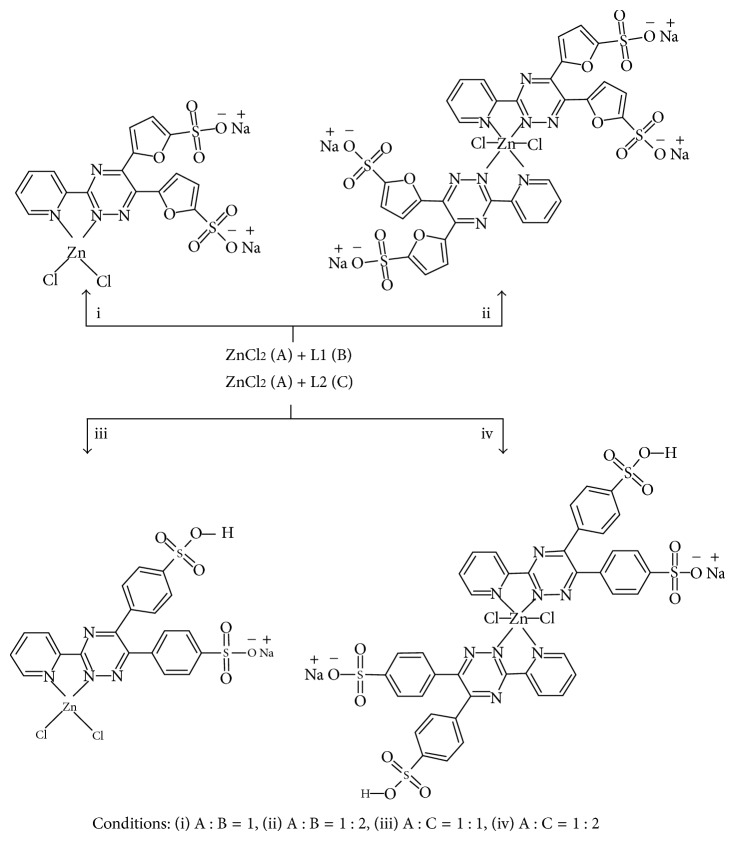
Synthetic routes for the preparation ML1Cl_2_ (complex **1**) (i), M(L1)_2_Cl_2_ (complex **2**) (ii), ML2Cl_2_ (complex **3**) (iii), and M(L2)_2_Cl_2_ (complex **4**) (iv) complexes. An associated molecule of ZnCl_2_ in complex **1** and solvent molecules in complexes **1–4** have been omitted for clarity.

Thus, we report here the synthesis and characterization of four novel metal complexes of the type ML_*n*_Cl_2_ (Figure 2) (where M = Zn^2+^, L = 3-(2-pyridyl)-5,6-di(2-furyl)-1,2,4-triazine-5′,5″-disulfonic acid disodium salt/3-(2-pyridyl)-5,6-diphenyl-1,2,4-triazine-4′,4″-disulfonic acid sodium salt and *n* = 1/2) and their BSA-binding studies.

## 2. Experimental

### 2.1. Materials and Methods

All chemicals (zinc chloride (3-(2-pyridyl)-5,6-di(2-furyl)-1,2,4-triazine-5′,5″-disulfonic acid disodium salt (ferene/L1) and 3-(2-pyridyl)-5,6-diphenyl-1,2,4-triazine-4′,4″-disulfonic acid sodium salt (ferrozine/L2)), methanol, diethyl ether, ethanol, bovine serum albumin (BSA), tris-HCl buffer (tris(hydroxymethyl)-aminomethane), sodium chloride (NaCl), and analytical grade water) were obtained from Sigma-Aldrich. All the solvents and chemicals were of analytical grade and were used as received, without further purification.

### 2.2. NMR Measurements

^1^H NMR spectra were recorded in D_2_O on a Bruker 400 MHz spectrometer. Peak positions are relative to tetramethylsilane (TMS) as reference. All NMR data were processed with TopSpin 3.2 and Mestre-C software.

### 2.3. Elemental Analysis

CHNS elemental analysis was performed by PerkinElmer 2400 Series II CHNS/O Elemental analyzer at Atlantic Microlab, USA.

### 2.4. Melting Point Determination

Melting points were manually determined in open capillaries.

### 2.5. UV-Visible Spectroscopy

Electronic spectra for ligand and metal complex were obtained on Spectro UV-Vis auto version 3.10, UV-2602 spectrophotometer. The spectral range was 200–800 nm. Spectra were obtained in methanol with baseline correction. Spectral data were processed with UV WIN software.

### 2.6. FTIR Analysis

FTIR spectra were recorded on a Thermo Scientific NICOLET iS10 spectrophotometer. ATR spectra were obtained within the 4000–600 cm^−1^ spectral range. Spectral data were processed with OMNIC software.

#### 2.6.1. Preparation of [Zn(L1)Cl_2_]·5H_2_O·ZnCl_2_ (1)

A solution of ferene (0.1 mmol, 0.0494 g) in methanol (4.0 cm^3^) was added to zinc chloride (0.1 mmol, 0.0140 g) in methanol (1.0 cm^3^). Then, the resulting mixture was stirred for 3 hours at 60–70°C [1]. The yellow precipitate which was obtained was collected by filtration, washed with ethanol and diethyl ether, and dried. C_16_H_8_Cl_2_N_4_Na_2_O_8_S_2_Zn·5H_2_O·ZnCl_2_, yield 0.0517 g, 78%. Anal. calc. for C_16_H_8_Cl_2_N_4_Na_2_O_8_S_2_Zn·5H_2_O·ZnCl_2_: C, 22.54; H, 1.92; N, 6.68; S, 7.64. Found: C, 22.13; H, 1.89; N, 6.57; S, 7.70%; melting point: >315°C; UV-Vis (MeoH) (*λ*_max_ (nm) (*ε* M^−1^·cm^−1^)): 210, 242, 331, 364; FTIR (ATR) (cm^−1^): 1504 (*υ*(N=N)),1582 (*υ*(C=N)); ^1^H NMR (D_2_O, *δ* ppm) 8.87 (d, H_6_), 8.79 (d, H_3_), 8.28 (t, H_4_), 7.87 (t, H_5_), 7.09–7.46 (m, 4H, furyl H).

#### 2.6.2. Preparation of [Zn(L1)_2_Cl_2_]·4H_2_O·2CH_3_OH (2)

A solution of ferene (0.2 mmol, 0.0988 g) in methanol (4.0 cm^3^) was added to zinc chloride (0.1 mmol, 0.0140 g) in methanol (1.0 cm^3^) [2]. Then, the resulting mixture was stirred for 6 hours at 60–70°C. The yellow precipitate which was obtained was collected by filtration, washed with ethanol and diethyl ether, and dried. C_32_H_16_Cl_2_N_8_Na_4_O_16_S_4_Zn·4H_2_O·2CH_3_OH, yield 0.0689 g, 61% based on zinc chloride. Anal. calc. for C_32_H_16_Cl_2_N_8_Na_4_O_16_S_4_Zn·4H_2_O·2CH_3_OH: C, 32.38; H, 2.56; N, 9.26; S, 10.57. Found: C, 32.5; H, 2.67; N, 9.63; S, 10.83%; melting point: >315°C; UV-Vis (MeoH) (*λ*_max_ (nm) (*ε* M^−1^·cm^−1^)): 209, 244, 337, 370; FTIR (ATR) (cm^−1^): 1511 (*υ*(N=N)),1586 (*υ*(C=N)); ^1^H NMR (D_2_O, *δ* ppm) 8.86 (d, H_6_), 8.76 (d, H_3_), 8.27 (t, H_4_), 7.84 (t, H_5_), 7.15–7.41 (m, 8H, furyl H).

#### 2.6.3. Preparation of [Zn(L2)Cl_2_]·3H_2_O·CH_3_OH (3)

A solution of ferrozine (0.1 mmol, 0.0492 g) in methanol (4.0 cm^3^) was added to zinc chloride (0.1 mmol, 0.0140 g) in methanol (1.0 cm^3^) [3]. Then, the resulting mixture was stirred for 3 hours at 60–70°C. The light yellow precipitate which was obtained was collected by filtration, washed with ethanol and diethyl ether, and dried. C_20_H_13_Cl_2_N_4_NaO_6_S_2_Zn·3H_2_O·CH_3_OH, yield 0.0433 g, 69% based on zinc chloride. Anal. calc. for C_20_H_13_Cl_2_N_4_NaO_6_S_2_Zn·3H_2_O·CH_3_OH: C, 35.28; H, 4.24; N, 7.84; S, 8.97. Found: C, 35.27; H, 4.36; N, 7.89; S, 9.13%; melting point: >315°C; UV-Vis (MeoH) (*λ*_max_ (nm) (*ε* M^−1^·cm^−1^)): 208, 240, 291, 323; FTIR (ATR) (cm^−1^): 1497 (*υ*(N=N)), 1599 (*υ*(C=N)); ^1^H NMR (D_2_O, *δ* ppm) 8.86 (d, H_6_), 8.76 (d, H_3_), 8.27 (t, H_4_), 7.84 (t, H_5_), 7.46–8.28 (m, 8H, phenyl H).

#### 2.6.4. Preparation of [Zn(L2)_2_Cl_2_]·5H_2_O·CH_3_OH (4)

A solution of ferrozine (0.2 mmol, 0.0984 g) in methanol (4.0 cm^3^) was added to zinc chloride (0.1 mmol, 0.0140 g) in methanol (1.0 cm^3^) [4]. Then, the resulting mixture was stirred for 6 hours at 60–70°C. The light yellow precipitate which was obtained was collected by filtration, washed with ethanol and diethyl ether, and dried. C_41_H_40_Cl_2_N_8_Na_2_O_18_S_4_Zn·5H_2_O·CH_3_OH, yield: 0.0579 g, 52% based on zinc chloride. Anal. calc. for C_41_H_40_Cl_2_N_8_Na_2_O_18_S_4_Zn·5H_2_O·CH_3_OH: C, 39.59; H, 3.24; N, 9.01; S, 10.32. Found: C, 38.93; H, 3.04; N, 8.84; S, 10.67%; melting point: >315°C; UV-Vis (MeoH) (*λ*_max_ (nm) (*ε* M^−1^·cm^−1^)): 216, 242, 298, 336; FTIR (ATR) (cm^−1^): 1498 (*υ*(N=N)), 1596 (*υ*(C=N)); ^1^H NMR (D_2_O, *δ* ppm) 9.05 (d, H_6_), 8.99 (d, H_3_), 8.67 (t, H_4_), 8.67 (t, H_5_), 7.50–8.31 (m, 16H, phenyl H).

### 2.7. BSA-Binding Assay

A 6 *µ*M BSA solution was prepared in a buffer containing 5 mM tris-HCl/50 mM NaCl by continuous stirring for 1 hr at room temperature. A 1 × 10^−3^ M stock solution of complexes and ligand was prepared in distilled water.

Absorption titration was carried out by keeping BSA concentration constant (6 *µ*M) and varying the concentrations of the complexes and ligand (010 *µ*M). After 10 min incubation at room temperature, absorbance was measured for each solution in the range of 250–300 nm wavelengths, and *λ*_max_ was recorded at 280 nm. Then, the change of *λ*_max_ was recorded for each solution. All absorbance measurements were triplicated and corrected for background absorbance by the compounds. The plot of 1/(*A *−* A*_0_) (where *A*_0_ is the initial absorbance of the free BSA at 280 nm and *A* is the absorbance of BSA in the presence of different concentrations of the complex) versus 1/[complex] is a linear curve, and the binding constant (*K*_b_) can be obtained from the ratio of the intercept to slope [19].

## 3. Results and Discussion

### 3.1. Synthesis

In order to synthesize the metal complexes, zinc chloride and the relevant ligands in 1 : 1 and 1 : 2 ratios were used (Figure 2).

### 3.2. UV-Visible Spectroscopy

UV-visible spectra of ligands and complexes **1–4** recorded in methanol showed significant differences between the absorption peaks of ligands and their complexes (Table 1, Figures \ S1 and S2, Supporting Information). Since both ligands bear conjugated systems, *π*–*π* ^∗^ transition is possible. In all four complexes, the wavelengths have shifted towards the longer wavelength range (bathochromic shift) because of changes in the conjugated electron system due to formation of metal ligand bonds. These observations are in agreement with previously reported zinc pyridyl triazine derivatives [18] and copper pyridyl triazine derivatives [22], upon coordination of ligand to metal.

### 3.3. ^1^H NMR Analysis

**Figure 3:**
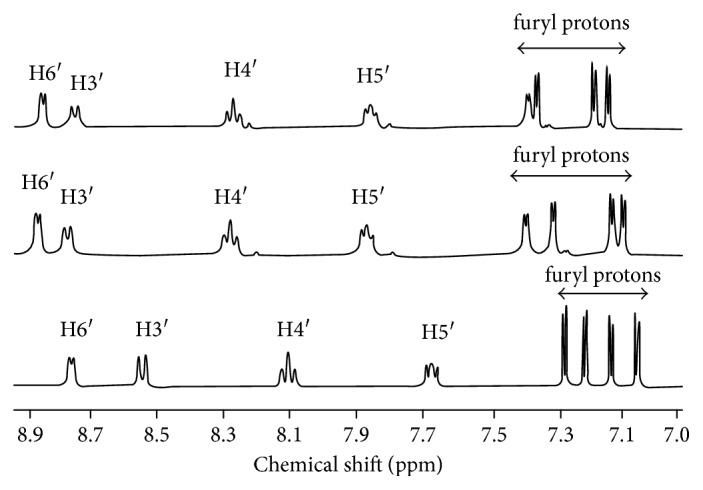
^1^H NMR spectrum of ferene (bottom) and complexes **1** (middle) and **2** (top).

**Figure 4:**
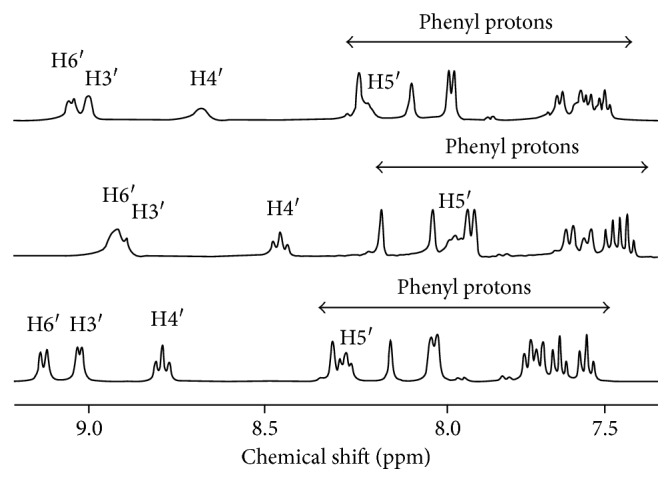
^1^H NMR spectrum of ferrozine (bottom) and complexes **3** (middle) and **4** (top).

Complexes **1–4** were characterized using ^1^H NMR spectroscopy in D_2_O. All the peaks were assigned in comparison with related structures of both ligands (Figures 3 and 4).

The splitting pattern of the free ferene ligand can be observed for complexes **1** and **2**. However, due to the donation of electrons from the nitrogen in pyridine and triazine rings to the metal, the electron density of the ferene ligand is reduced, and thus protons of the metal complex should appear more downfield than the ferene ligand. Furthermore, the downfield shift, which will hence be denoted as ∆*δ*, of the H_6_ signal is expected to be higher than that of other protons because it is closer to the pyridine N. In uncoordinated ferene ligand (L1), the pyridyl H_6_ signal (8.74 ppm, Table 2) is its most downfield doublet consistent with its close proximity to the pyridyl nitrogen atom. In a spectrum of complex **1**, the H_6_ signal appears further downfield (8.87 ppm, Figure 3) confirming metal-pyridine N bond formation. However, the observed change in shift of H_6_ was small in both **1** (∆*δ*; +0.13 ppm at 8.87 ppm) and **2** ((∆*δ*; +0.12 ppm at 8.86 ppm) in comparison with the change in shift observed for the H_3_ proton (8.79 ppm and 8.76 ppm), which had the highest change in downfield shift ((∆*δ*; +0.25 and +0.22 ppm, resp.). The four doublets due to furyl ring protons (7.00–7.34 ppm range) also appear more downfield (7.15–7.45 ppm) upon metal bonding. Higher downfield shifts were observed for H_3_ versus H_6_ in ^1^H NMR spectra of Zn(dppt)Cl_2_·0·5H_2_O and Zn(dppt)_2_Cl_2_·2H_2_O (dppt = 5, 6 diphenyl-3-(2-pyridyl)-1,2,4- triazine) reported previously [18].

Although we expected similar observations for spectra of complexes **3** and **4**, unusual upfield shifts of the peaks attributed to H_6_ (8.92 and 9.05 ppm), H_5_ (7.98 and 8.21 ppm), H_4_ (8.47 and 8.67 ppm), and H_3_ (8.89 and 8.99 ppm) (Figure 4) were observed in complexes **3** and **4**, respectively, in comparison with that of the uncoordinated ferrozine ligand (H_6_: 9.15 ppm, H_5_: 8.27 ppm, H_4_: 8.81 ppm, and H_3_: 9.05 ppm). We attribute the observed upfield shift to possible *π* stacking of phenyl rings. Upfield shifts due to *π* stacking have been reported in previous studies on Zn(II) with mono- and dianionic pyrrole-2-imine complexes and zinc azadipyrromethene [23]. Although the ^1^H NMR spectrum of ferrozine/L2 is comparatively more complicated due to the protons of the two phenyl rings, the pyridyl ring protons can be easily distinguished (Figure 4). Upfield shifts observed in complexes **3** and **4** were not observed in complexes **1** and **2**, which had furyl rings (Figure 3).

### 3.4. FTIR Analysis

Literature data have been used where relevant to get assignment of ligands [18]. The stretching frequency (*ѵ*) of the N=N bond in the triazine ring and C=N bond in the pyridine ring serves as important indicators of the formation of new metal ligand bonds [18]. In all four complexes, *ν*_N=N_ and *ν*_C=N_ have shifted to lower frequencies (Table 3) due to the formation of new metal ligand bonds which in turn lowers the strength of the N=N and C=N bonds. This observation allowed us to confirm that the complex was formed via the donation of a lone pair of electrons each, from the triazine ring and from the pyridine ring, to zinc. For example, upon formation of complex **1**, *ν*_C=N_ (1590 cm^−1^) and *ν*_N=N_ (1510 cm^−1^) appear at 1582 cm^−1^ and 1504 cm^−1^ (Table 3), respectively, due to the change in chemical environment. FTIR spectra show broad peaks at around 3400 cm^−1^ region due to OH vibration.

### 3.5. Elemental Analysis

Empirical formulas related to experimental values of the complexes (Experimental) give the exact molecular formulas and experimental values are closer to the expected values. Some deviate from the theoretical values due to the residual solvent (methanol) and water molecules.

Elemental analysis data suggest that complex **1** exists as [Zn(L1)Cl_2_]·5H_2_O·ZnCl_2_. The experimental data obtained from elemental analysis show a significant decrease in the carbon percentage of this complex than expected without the extra zinc ion, prompting us to include an associated zinc chloride molecule in **1**. However, the exact binding mode or type of the interaction of this extra ZnCl_2_ molecule with ferene ligand cannot be explained with the obtained data. Complexes **2–4** have no such discrepancy, and the molecular formula were confirmed from elemental analysis to be [Zn(L1)_2_Cl_2_]·4H_2_O·2CH_3_OH, [Zn(L2)Cl_2_]·3H_2_O·CH_3_OH, and [Zn(L2)_2_Cl_2_]·5H_2_O·CH_3_OH, respectively.

### 3.6. BSA-Binding Assay

Anjomshoa and coworkers have previously investigated bovine serum albumin- (BSA-) binding properties of Zn(dppt)_2_Cl_2_·2H_2_O (dppt = 5,6-diphenyl-3,2-pyridyl-1 2,4-triazine) [19], which required an organic solvent such as dimethyl sulfoxide to be added to increase solubility. However, complexes **1–4** reported in this study are highly soluble in water which makes them compatible with biological systems.

**Figure 5:**
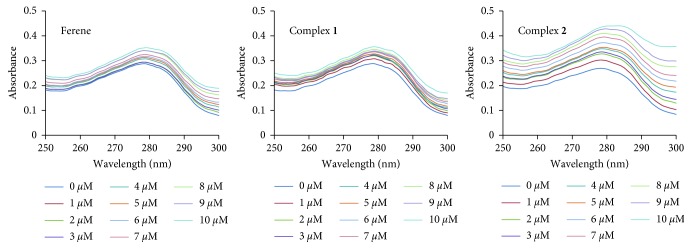
Absorbance spectra of BSA in the presence of different concentrations of ferene, complex **1**, and complex **2**.

Absorbance measurements at UV range are useful to identify the conformational changes in proteins. BSA has a maximum absorbance peak at 280 nm. Analyzing the absorbance spectra of BSA upon addition of ferene, complex **1**, and complex **2** (Figure 5) clearly shows that the peak intensity at 280 nm increases upon addition of the compounds, indicating association with BSA causes a change in the polarity of the microenvironment of tryptophan and tyrosine residues in a concentration dependent manner.

**Figure 6:**
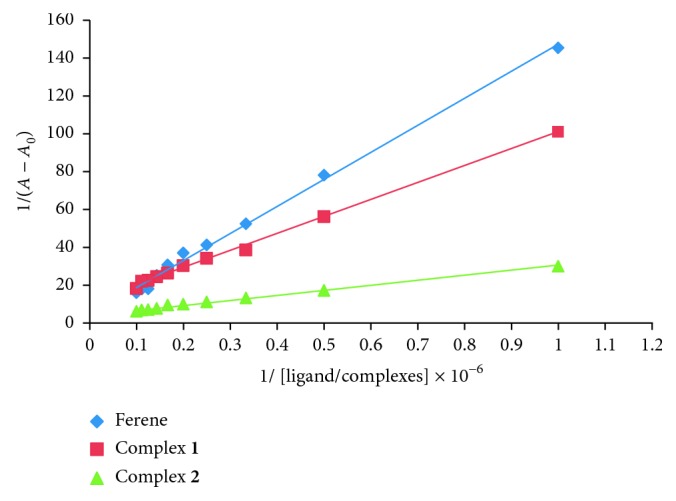
The graph of 1/(*A *−* A*_0_) versus 1/[ligand/complexes] for ferene ligand (*R* ^2^ = 0.9955), complex **1** (*R* ^2^ = 0.9951), and complex **2** (*R* ^2^ = 0.9955).

With increasing concentrations of ferene ligand and complexes **1** and **2**, the maximum absorbance was increased. Using the graph of 1/(*A *−* A*_0_) against 1/[C], binding constants were calculated (Figure 6) as 3.09 × 10^4^ M^−1^, 12.30 × 10^4^ M^−1^, and 16.84 × 10^4^ M^−1^ for ferene, complex **1**, and complex **2**, respectively. These values are within the range of 10^4^–10^6^ M^−1^ as expected from a good BSA carrier activity *in vivo* [19]. Such changes upon BSA addition were not observed in ferrozine and complexes **3** and **4**.

## 4. Conclusions

In this study, we have described the synthesis and characterization of four novel zinc complexes by spectroscopic methods. According to the UV-Vis spectra, a bathochromic shift has been observed for all four complexes. FTIR data provide evidence that Zn-N bonds are formed via N atoms of the triazine and pyridine rings. Accordingly, the stretching frequency of N=N and C=N bonds of all four complexes have shifted to the low frequency range in comparison with the free ligands. Elemental analysis was used to determine the empirical formula of complexes **1–4**.

It is our belief that the scaffolds reported herein may provide a novel platform for drug designing. We have demonstrated that such systems possess high affinity to serum albumin, indicating their potential to be distributed in serum [24]. Sulfonated groups have aided to increase water solubility of the complexes, where aqueous solubility predicted by ChemAxon (https://disco.chemaxon.com) have also supported that the new complexes are soluble in water under biologically relevant pH ranges (data not shown). *In vivo* testing is warranted to explore the implications of such properties on cellular metabolism to delineate the function of the novel triazine complexes in biological systems.

## Figures and Tables

**Table 1:** Comparison of UV-Vis data of ferene, ferrozine, and complexes **1–4**.

*λ*_Zn^2+^_ (nm)	*λ*_ferene_ (nm)	*λ*_complex **1**_ (nm)	*λ*_complex **2**_ (nm)	*λ*_ferrozine_ (nm)	*λ*_complex **3**_ (nm)	*λ*_complex **4**_ (nm)
207	209	210	209	211	208	216
222	243	242	244	236	240	242
—	306	331	337	285	291	298
—	339	364	370	310	323	336

**Table 2:** Selected ^1^H NMR chemical shifts (ppm) of ferene, ferrozine, and complexes **1–4**.

Proton no.	H_6_ (d)	H_5_ (t)	H_4_ (t)	H_3_ (d)
Ferene	8.74	7.67	8.10	8.54
Ferrozine	9.15	8.27	8.81	9.05
Complex **1**	8.87	7.87	8.28	8.79
Complex **2**	8.86	7.84	8.27	8.76
Complex **3**	8.92	7.98	8.47	8.89
Complex **4**	9.05	8.21	8.67	8.99
∆*δ* (ppm) of complex **1**	(+) 0.13	(+) 0.20	(+) 0.18	(+) 0.25
∆*δ* (ppm) of complex **2**	(+) 0.12	(+) 0.17	(+) 0.17	(+) 0.22
∆*δ* (ppm) of complex **3**	(−) 0.23	(−) 0.29	(−) 0.34	(−) 0.16
∆*δ* (ppm) of complex **4**	(−) 0.10	(−) 0.06	(−) 0.14	(−) 0.06

**Table 3:** FTIR data of complexes **1–4**.

Ligand/complex	*ν*_C=N_ (cm^−1^)	*ν*_N=N_ (cm^−1^)
Ferene	1590	1510
Complex **1**	1582	1504
Complex **2**	1586	1511
Ferrozine	1608	1502
Complex **3**	1599	1497
Complex **4**	1596	1498

## Supplementary Materials

Supplementary Materials Figure S1. UV-visible spectra of ZnCl_2_, ferene, and complexes **1** and **2**. Figure S2. UV-visible spectra of ZnCl_2_, ferrozine, and complexes **3** and **4**.
