# Supplementary material for: Synthesis, Characterization, and BSA-Binding Studies of Novel Sulfonated Zinc-Triazine Complexes
Source: Bioinorg Chem Appl. 2018 Feb 18;2018:7563820. doi: 10.1155/2018/7563820 (PMC5835287; doi:10.1155/2018/7563820)
Supplement: Supplementary Materials — Figure S1. UV-visible spectra of ZnCl2, ferene, and complexes 1 and 2. Figure S2. UV-visible spectra of ZnCl2, ferrozine, and complexes 3 and 4. [file 7563820.f1.pdf]

Supporting Information

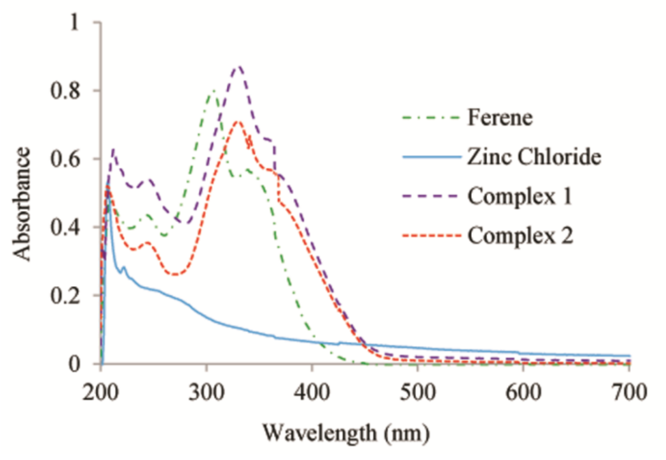

FIGURE S1. UV-Visible spectra of $ZnCl_2$, ferene, and complexes **1** and **2**

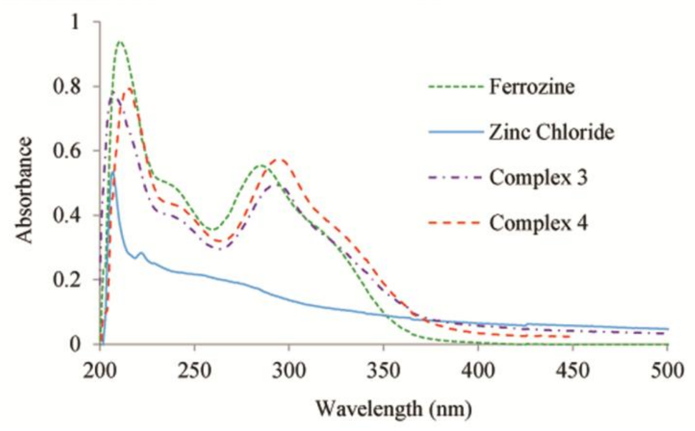

FIGURE S2. UV-Visible Spectra of $ZnCl_2$, ferrozine, and complexes **3** and **4**
